# Global Scientific Trends on Healthy Eating from 2002 to 2021: A Bibliometric and Visualized Analysis

**DOI:** 10.3390/nu15061461

**Published:** 2023-03-17

**Authors:** Te Fang, Hongyi Cao, Yue Wang, Yang Gong, Zhongqing Wang

**Affiliations:** 1 Department of Anesthesiology, The First Affiliated Hospital of China Medical University, Shenyang 110001, China; 2 Department of Pathology, College of Basic Medical Science, China Medical University, Shenyang 110001, China; hycao0222@126.com; 3 Department of Clinical Nutrition, The First Affiliated Hospital of China Medical University, Shenyang 110001, China; 4 School of Biomedical Informatics, The University of Texas Health Science Center at Houston, Houston, TX 77030, USA; 5 Department of Information Center, The First Affiliated Hospital of China Medical University, No.155, Nanjing Street, Heping District, Shenyang 110001, China

**Keywords:** bibliometrics, VOSviewer, healthy eating, global trend, sustainability

## Abstract

Diet has been recognized as a vital risk factor for non-communicable diseases (NCDs), climate changes, and increasing population, which has been reflected by a rapidly growing body of the literature related to healthy eating. To reveal a panorama of the topics related to healthy eating, this study aimed to characterize and visualize the knowledge structure, hotspots, and trends in this field over the past two decades through bibliometric analyses. Publications related to healthy eating between 1 January 2002 and 31 December 2021 were retrieved and extracted from the Web of Science database. The characteristics of articles including publication years, journals, authors, institutions, countries/regions, references, and keywords were assessed. The analyses on co-authorship, co-occurrence, and co-citation were performed and network visualization maps were constructed by VOSviewer. Major subdomains identified by bibliometrics were further discussed and analyzed. A total of 12,442 articles on healthy eating were identified. Over the past two decades, the annual global publications increased from 71 to 1764, showing a nearly 25-fold growth. The journal *Nutrients* published the most articles and *The American Journal of Clinical Nutrition* possessed the highest citations. The United States, Harvard University, and Hu, Frank B. were identified as the most productive and influential country, institution, and author, respectively. The co-occurrence cluster analysis of the top 100 keywords formed four clusters: (1) the food insecurity environment for youths highlighting the necessity and significance of implementing healthy eating in early life; (2) sustainable advantages of the Mediterranean diet; (3) the importance of an overall healthy lifestyle optimization leveraged by eHealth; (4) the challenges during the course of healthy eating against obesity, which are prominent in reflecting the knowledge structure, hotspots, and trends. Moreover, COVID-19, orthorexia nervosa, sustainability, microbiota, food insecurity, and e-health are identified keywords that represented the latest high-frequency keywords and indicated the emerging frontiers of healthy eating. This study indicates that the number of publications on healthy eating will increase in the future and that healthy dietary patterns and clinical applications of healthy eating will be the next hotspots in this research field.

## 1. Background

Climate change induced by global agriculture, increasing population, and the epidemic of non-communicable diseases (NCDs) are the global major challenges [1] that have established a tightly linked population–environment–health trilemma and present strong interactions often driven by dietary changes [2]. Healthy eating is regarded as a vital alleviator of confronting the three challenges, as diets are modifiable [2].

Global agriculture has substantial impacts on the environment, reflecting in occupying around half of the ice-free land surface, polluting cropland, pastureland, and water with agrochemicals, and threatening species with extinction by clearing tropical forests, savannas, and grasslands [3]. Nowadays, agriculture accounts for over 30% of total greenhouse gas emissions and is nominated as a pivotal contributor to climate change, as inherently rooted in the way of cultivation, production, processing, distribution, consumption, and wasting [4]. Despite the barbarous development of global agriculture, a billion people still suffer from starvation and insecure food supplies [5]. According to the Food and Agriculture Organization prediction, there will be an increase of over 60% in global food production to feed the growing world population, namely over nine billion people by 2050 [6], which poses a great challenge to the simultaneous preservation of human and planet health. Moreover, fast urbanization is driving a global dietary shift towards westernized patterns, where traditional diets are replaced by food higher in refined cereals, oils, red meats, and ultra-processed foods [7]. Such dietary transition, if not intervened, would become a dominant contributor to an estimated 80% increase in global agricultural greenhouse gas emissions by 2050 [1]. Additionally, current dietary trends are greatly raising the incidence of NCDs, which account for up to 72% of worldwide mortality and greatly contribute to the global burden of disease [8], where dietary factors are a major contributor and leading risk among NCDs for death in adults (22%) [9]. Extensive studies assessing associations between dietary habits and NCDs revealed a causal relationship between increasingly westernized dietary patterns and the marked rise in NCDs [10].

Although healthy eating plays a pivotal role in counteracting the negative consequences of the aforementioned three challenges, the implementation of healthy eating is highly complex concerning the interplay of human, socioeconomic, environmental, and political factors. For such complexity, evidence-based definitions of healthy eating, efficient assistant strategies for generalization, and policy benefit for extensive popularization are highly warranted. To our best knowledge, research in the field of healthy eating has not yet been systematically reviewed. Bibliometrics, a cross-discipline analysis to mine and obtain comprehensive knowledge information in a certain domain by leveraging mathematics, statistics, and philology [11], has been widely applied to analyze quantitative changes, knowledge structures, collaboration strength, research hotspots, and development trends in research domains [12]. In contrast to traditional analysis methods, bibliometrics has the potential to visualize the “big picture” of extant research on a given topic [13].

This bibliometric study aimed to explore the evolution of healthy eating research over the past two decades, identifying and describing the current conception of healthy eating, examining the values and bugs during its application, and predicting research trends. This review has not only important theoretical and methodological enlightenments for researchers and personalized suggestions for individuals but is also particularly informative to policy regulators and makers. This bibliometric study is expected to substantially update and promote healthy eating and respond to global challenges. The findings of this analysis hold the potential to serve as a general framework, spark and guide research in the healthy eating area, and facilitate global collaborations in the rapidly developing field of healthy eating.

## 2. Methods

### 2.1. Data Source

The Web of Science (WoS), incorporating over 9000 academic journals across extensive scientific fields, is a large and comprehensive academic information platform in the world [14]. The WoS that fulfills all the basic requirements for bibliometric analysis [15] was employed as a data source to retrieve the relevant publications in this study.

### 2.2. Search Strategy

**Figure 1:**
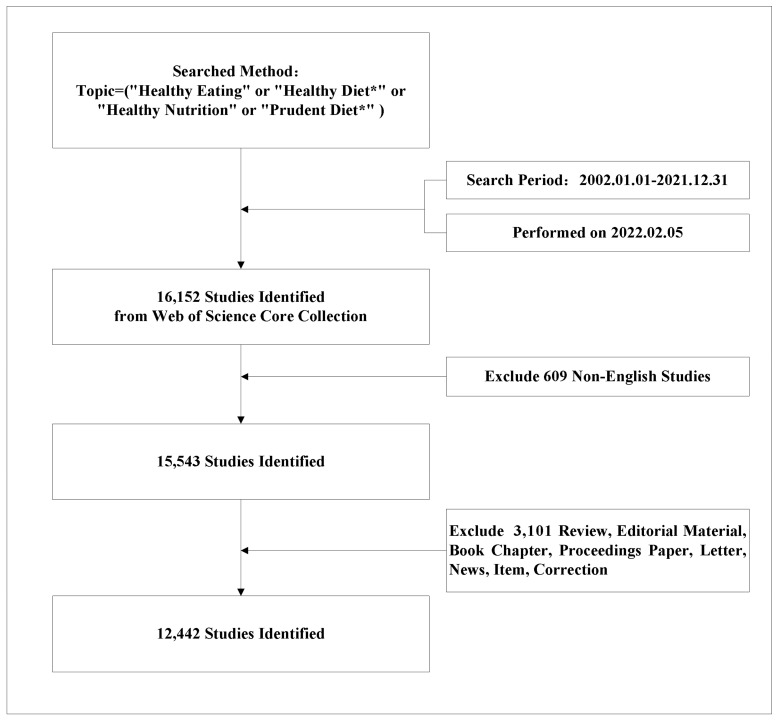
The flowchart of the literature search process.

The literature retrieval process is illustrated in Figure 1. Publications on the topic of healthy eating between 1 January 2002 and 31 December 2021 were searched on the WoS core collection. The search strategy was as follows: topic search (TS) = (“Healthy Eating” or “Healthy Diet*” or “Healthy Nutrition” or “Prudent Diet*”). The publication language was limited to English. The publication type was set to articles only. The information of publications, including the title, publication year, author, country/region, institution, journal, keyword, and abstract, were downloaded in the form of TXT files. Both searches and downloads were carried out on 5 February 2022 to avoid the impact of frequent updates of the WoS database.

### 2.3. Patient and Public Involvement

Patients were not involved in this study.

### 2.4. Data Analysis

VOSviewer1.6.18, analysis software developed based on the JAVA platform [16], was employed to visualize the evolution path, distribution structure, collaboration, and frontier in the healthy eating research by analyzing publishing volume, cooperation of authors and institutions, co-citation of journals, and co-occurrence of keywords. The major topics relating to healthy eating were identified using both co-occurrence network maps and overlay maps of keywords. In the network map, the nodes in graphs are labeled by the objects of the analysis and colored. The node size represents the frequency of citation or occurrence. The thickness and length of links between nodes represent the strength of collaboration. In the overlay map, the color of nodes represents the average publication year of the keywords. The color shift from blue to red corresponds to the average publication year from the initial to the latest. Furthermore, descriptive analyses of publications, including publication years, journals, countries/regions, institutions, authors, and references, were performed to identify active and prolific individuals and teams. In addition, the gender of prolific authors and the journal quartiles of productive journals based on Journal Citation Reports (JCR) were analyzed.

## 3. Results

### 3.1. Global Publishing Volume and Trend

**Figure 2:**
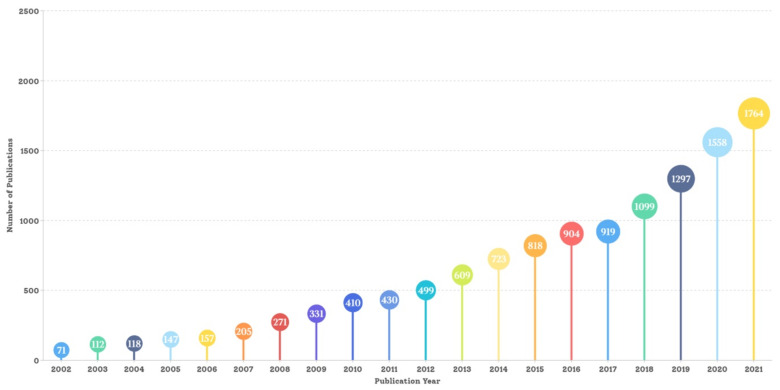
Annual global publications on healthy eating research between 2002 and 2021.

A total of 12,442 publications were identified from the WoS core collection. Figure 2 shows that the number of annual publications increased from 71 in 2002 to 1764 in 2021, exceeding 1000 in 2018. There has been approximately a 25-fold increase in the publishing volume over the past 20 years. Additionally, the number of annual publications presented an increasing trend, doubling the volume every 5 years. A total of 6637 articles were published from 2017 to 2021, accounting for 53.34% of the total volume over the two decades.

### 3.2. Distribution and Contribution of Countries/Regions

Globally, 153 countries/regions have carried out research on healthy eating. Supplementary Figure S1 shows the distribution and contribution of the 32 most productive countries/regions with publications over 100. Among them, three countries/regions had published more than 1000 articles on this topic, which contributed to 60.5% of total publications. The amounts of publications from the other 150 countries/regions merely occupied less than 40%. Over half of the productive countries/regions were located in Europe, followed by Asia (25%), and others scattered in North America and Oceania, whereas only one productive country/region was located in Africa and South America, respectively. 

Supplementary Table S1 shows the top 10 most productive countries/regions. In terms of the total number of publications and citation frequency, the United States topped the list (4822 counts and 129,710 citations), followed by the United Kingdom (1418 counts and 48,000 citations) and Australia (1284 counts and 33,901 citations). Among them, Germany had the highest average citations. As per the latest average publication year, China and Brazil occupied the first (2017.82) and second (2017.25), respectively.

### 3.3. Contribution of Journals

A total of 1926 journals published articles on the topic of healthy eating. Supplementary Table S2 shows the top 10 most productive journals. The 3096 articles documented in the top 10 journals accounted for 24.88% of the total publications. The most productive journal on this topic was *Nutrients* (678), followed by *Public Health Nutrition* (475) and *Appetite* (399). *The American Journal of Clinical Nutrition* ranked first in terms of influence factor (7.047), citation frequency (12,121), and average citations (60). However, *International Journal of Environmental Research* and *Public Health* had the latest average publication year (2019.50), followed by *Nutrients* (2019.36). Among them, four top productive journals belonged to the United States and the other six were published in Europe. Based on JCR reported in 2022, four journals were grouped in Quartile 1 (Q1), five belonged to Quartile 2 (Q2), and one was in Quartile 3 (Q3). 

Scientific research is typically based on the evidence and lessons learned from prior studies. The analysis of cited references in the retrieved articles is expected to help researchers understand the foundations, challenges, and opportunities in this field. A total of 310,664 references gleaned from 69,876 journals were cited by the retrieval articles on the topic of healthy eating. Among 69,876 journals, 69 journals had been cited at least 1000 times. A co-citation analysis of 69 highly cited journals and the network visualization map constructed by VOSviewer formed three clusters (Supplementary Figure S2). *The American Journal of Clinical Nutrition*, *Journal of the American Dietetic Association*, *Public Health Nutrition*, and *Appetite* were the top four most co-cited journals with co-citation rates of over 10,000 in the network map.

### 3.4. Contribution and Cooperation of Institutions

As per the publications, 9943 institutions were involved in the research of healthy eating. Supplementary Table S3 lists the top 10 most productive institutions. Half (n = 5) of the institutions were located in the United States. Two institutions were located in Australia and one in Canada, Brazil, and Sweden, respectively. Harvard University published the most papers on this topic (477), followed by Deakin University (271) and the University of Minnesota (228). The citation frequency of Harvard University ranked first (23,020), followed by Brigham and Women’s Hospital (11,220) and the University of Minnesota (10,183). The citation frequency of all seven other institutions is no greater than 9000. Brigham and Women’s Hospital had the highest average citations (67), followed by Harvard University (48) and the University of Minnesota (45). The University of Sao Paulo had the latest average publication year (2017.16), the second was Karolinska Institute (2016.44), and Tufts University (2016.20) and Harvard University (2016.16) occupied the third and fourth, respectively.

Out of the 9943 institutions, 153 were identified surpassing a threshold of 40 publications. The co-authorship network map of the 153 institutions consists of seven clusters (Supplementary Figure S3). Four academic groups were located in North America, two in Europe, and one in Australia. The red cluster represented the biggest academic cooperative group, including 40 research institutions. Closely internal and external cooperation were found among three American groups (red, purple, and orange clusters). However, the cooperation ties were relatively weak and inconsistent within and across the other four groups. The top 10 productive institutions were also the academic cores of the seven groups. Harvard University is shown as a core collaborating with other 122 productive institutions and became a prominent hub of the overall network, followed by the University of Washington (86) and Tufts University (83).

### 3.5. Contribution and Cooperation of Authors

A total of 49,761 researchers have published articles on the topic of healthy eating. Supplementary Table S4 lists the top 10 researchers who have contributed greatly to this field. Among them, seven authors were affiliated with American academic institutions and the other three were from Australia. Additionally, seven authors were male and the other three were female. Hu, Frank B. was the most productive author (75) and Willett, Walter C. ranked second (65), followed by Pierce, John P. (48). The publications of Hu, Frank B. were cited mostly (7526), followed by Willett, Walter C. (4795) and Rimm, Eric B. (3159). With respect to average citations, Hu, Frank B. was the first (100), followed by Rimm, Eric B. (81) and Fung, Teresa T. (75). Drewnowski, Adam possessed the latest average publication year (2017.23) and Rimm, Eric B. ranked second (2017.03). In terms of the number of publications, citation frequency, average citations, and average publication year, the authors who topped the list were all from the US.

By setting a threshold of 10 publications, 374 out of the 497,613 authors were identified as productive authors. Among them, 324 productive authors constructed a co-authorship network that comprises 15 clusters (Supplementary Figure S4). Hu, Frank B. cooperated with 54 other productive authors, followed by Manios, Yannis (39) and Moschonis, George (35). Notably, four groups (yellow, orange, green, and grey) were extremely active in terms of publications, which is reflected by a stronger cooperative link within the groups.

### 3.6. Analysis of Keywords and Research Trend

**Figure 3:**
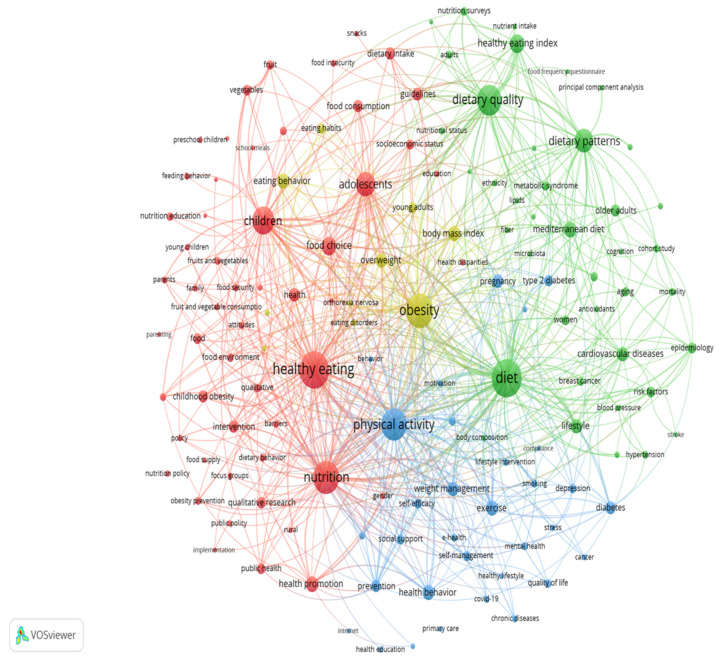
The co-occurrence network map of 137 high-frequency keywords.

Among 15,843 keywords originating from the 12,422 articles included in this study, 137 high-frequency keywords were identified and extracted based on a threshold of 50. Excluding the central keyword “healthy eating”, the top five high-frequency keywords were identified as diet (1173 counts), obesity (1004 counts), nutrition (939 counts), physical activity (896 counts), and dietary quality (816 counts). The 137 high-frequency keywords exhibited a co-occurrence network comprising four clusters (Figure 3). Group 1 (red cluster) was the biggest cluster consisting of 54 keywords. Group 2 (green cluster) ranked second, followed by Group 3 and Group 4 (blue and yellow clusters).

**Figure 4:**
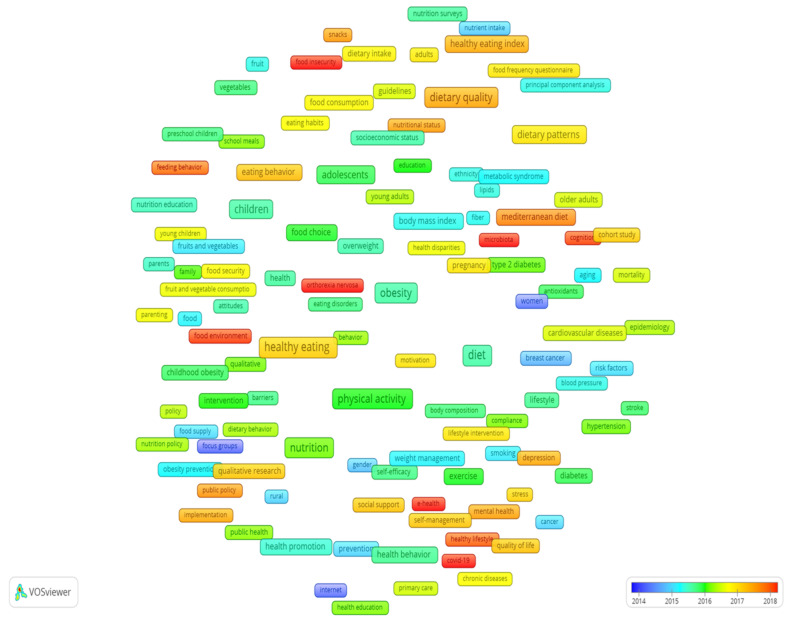
The overlay map of the 137 high-frequency keywords.

The overlay visualization map was used to monitor the research trend in this field (Figure 4). The keywords with the latest average publication year were identified as COVID-19 (2020.76), orthorexia nervosa (2019.08), sustainability (2019.01), microbiota (2018.40), food insecurity (2018.25), and e-health (2018.17).

## 4. Discussion

### 4.1. Global Publishing Trends

The variation of the publishing volume reflects the development trend in the academic field. The articles on the topic of healthy eating were authored by over 70% of countries/regions around the world, exhibiting an exponential growth of the body of the literature. The data analyzed in this research indicate that the field of healthy eating is attractive and promising; more in-depth studies with novel perspectives on healthy eating are expected in the future.

### 4.2. International Research Status and Cooperation

The publishing volume and corresponding cited frequency represent the academic position and influence of a country/region. Europe represents the most significant contributor to the field of healthy eating research, followed by North America, Asia, and Oceania. Of the publications identified, nearly 40% were published in the United States and over 10% in the United Kingdom and Australia, respectively, which are the three leading countries in this research field. However, it is observed that the productive regions in Africa and South America are scarce. This kind of uneven distribution in research activities on the topic may be influenced by the economic status [17]. The diet in most developed and industrialized countries has already exceeded the recommended intake of energy per nutritional guidelines in terms of increasing the consumption of sugar, fat, and protein [18]. Therefore, the nutritional issues initially were observed to affect developed countries/regions, such as European and North American countries/regions. The prevailing western dietary patterns have caused substantial challenges and serious problems concerning increasing healthcare demands and irreversible environmental deterioration, which might have driven research to alleviate dietary adverse influences through early interventions. Meanwhile, as developing countries, China and Brazil have been experiencing a dramatic nutrition transition driven by fast economic development and urbanization [19]. As a result, Both China and Brazil exhibited rapid growth in publications on this topic. Given the high density of populations and the prevalence of diet-related NCDs in these two countries [20], a deeper understanding of the role that healthy eating may play in shaping physical conditions is warranted.

Although the United States has contributed the most publications in this field, its average citation is notably lower than that of Germany. Nutrition research in Germany focusing on the molecular mechanisms revealed the correlation between risks of diseases and the level of animal and human food exposure, demonstrating a strong impact reflected by citations [21,22].

The co-authorship network map that has enrolled the most prolific institutions and authors implies that this research field has increasingly gained much attention and collaboration. The networks represented in the maps consist of numerous collaborative groups of various sizes and strengths. The connections within and across the three American research groups are active and profound; however, the cooperative ties across the other teams are generally weak and inconsistent, inferring that the United States attached great importance to exchanges in this academic community and to some extent explained the reason why the United States has such a high output in this field. The top 10 productive institutions and authors were almost at the cores of each cooperative group; among them, Harvard University and Hu, Frank B. were the global most prestigious institution and author, respectively. Moreover, most of the top 10 prolific authors were male. Additionally, among authors or institutions with relevant works in this field only nearly 0.1% were identified as prolific, indicating that the majority of academic groups or individuals were passers or beginners, who were interested in engaging with this research but their research continuity and opportunities for cooperation remained limited. Therefore, healthy eating research is at an early stage of fast development. Overall, cooperation in this field should be enhanced to provide an affluent platform for sharing experience and advancement, to expand research scale and scope, to resolve more academic issues, and to thus improve the deep-going of articles.

The analyses of contribution of journals, spatial distribution of the literature, preferences of journals published on this topic, and core and marginal journals are used to offer researchers reliable references to determine on which journals the articles published they should mainly focus and where to submit their research accomplishments. The top 10 journals published approximately 25% of total publications in this field and were distributed in high-quality journal quartiles (Q1 and Q2), demonstrating “healthy eating” is a hot and promising topic and that the evidence of this review is robust and compelling. Among them, four were founded by the United States and six by several European countries. *The American Journal of Clinical Nutrition*, *Public Health Nutrition*, and *Appetite* are the core journals with relatively high publishing capacity and influence in this field. *Nutrients* has become the dark horse with the largest and latest publications. Overall, the top 10 journals are prone to provide the main access to vital and groundbreaking information in the future.

### 4.3. Hot Spots and Novel Themes

Keywords are used to summarize the main theme and research method of the literature. Accordingly, keywords with high frequency are often used to identify major topics, generate knowledge foundation and structure, and search for cutting-edge perspectives in research domains. 

A co-occurrence network map of high-frequency keywords was generated in four clusters (Figure 3). The density of the entire network indicates that most of the literature in this field is multi-faceted research. Based on the frequency and centrality, four clusters were analyzed as below.

Cluster 1 (red) mainly focuses on that the current food insecurity environment for youths sheds light on the necessity and significance of implementing healthy eating in early life. Nutrition in early life plays a vital role in lifelong health [23]. Youth is an early life stage characterized by the rapid development of organs and systems. Variation in both quality and quantity of youth nutrients can exert permanent and powerful effects on the growth and development [23]. These effects represent an important risk factor for NCDs in adulthood, which can be transmitted to further generations by heritable epigenetic modifications [24]. The youths aged 0-19 account for over one-third of the global population [25], but the youths are growing up in an insecure food environment with both persisting nutrition deficiency and widespread obesity [23]. Currently, major concerns are the excessive consumption of ultra-processed foods (UPFs) and take-away food [10]. UPFs contribute 25–67% of the total energy among youths [10,26]. Although the data on the consumption of take-away food remain unclear, the COVID-19 pandemic lockdown policy has led to a rapid expansion in its consumption [27,28]. Both UPFs and take-away food are commonly high in energy density, sugars, salt, trans fats, additives, and chemical contaminants but low in protein, micronutrients, and fibers [29,30] and are confirmed to increase the risk of NCDs among youths [31,32]. Additional evidence demonstrated that 15% of youths showed addictive-like responses to UPFs, defined as food addiction [33,34], sharing similar functional and structural changes in neurological systems with drug abuse [35]. In this context, policies or strategies implemented by governments, such as warning labels on packages and additional taxes, do not have the intended effect of decreasing unhealthy food purchasing, because most parents either ignore them or fail to understand their significance [36]. The integration of empirical findings encourages healthy eating practices among children supported by their parents via the following persuasive strategies: positive parental, eating together, healthy home food environments, creating the pleasure of eating, shaping attitudinal, intentional, and behavioral outcomes in children [37]. The current insecure food environment for youths set off an alarm to the policymakers, monitoring departments, and guardians that implementation of healthy eating in youths is imperative, which would define the rate of morbidity and mortality of NCDs in the present and next generations. 

Cluster 2 (green) mainly focuses on the sustainable advantages of the Mediterranean diet. Conventional research on healthy eating merely focused on the health benefits of nutrients and dietary patterns [38]; nowadays, the updated conception of healthy eating has been proposed as nutritionally adequate, safe, healthy, culturally acceptable, economically affordable, and environmentally friendly [39,40]. Thus, sustainable aspects are needed to be incorporated into healthy eating. For example, the Mediterranean diet (MedDiet) fulfilled the criteria and has gained much attention recently [40]. Concerning nutrition and health benefits, MedDiet largely consists of fresh vegetables and fruits, fish, grains, legumes, olive oil [41], all of which are rich in unsaturated fatty acids [42], fiber [43], high-quality plant protein [44,45,46], antioxidants [47], and anti-inflammatory chemicals [48]. The favorable effects of MedDiet on health may depend on the positive impact of the aforementioned nutrients on the composition, function, and production of gut microbiota [49,50]. In terms of lower environmental impacts, MedDiet has evident advantages as a plant-based diet with minimal carbon and water footprint [2]. Additionally, MedDiet can promote richness in biodiversity by facilitating the consumption of local, seasonal, and traditional food [40]. Regarding high socio-cultural values, MedDiet was acknowledged as an Intangible Cultural Heritage of Humanity in 2010 [51]. Rather than physiological needs, meals in the MedDiet culture are a moment of pleasure that represents a daily opportunity for social exchange and communication [52]. Moreover, frugality is embodied in MedDiet evidenced by food preparation, moderate portion size, and avoiding waste [52]. In terms of positive local economic returns, MedDiet could promote the consumption of fresh food available locally and thereby produce economic benefits by reducing agricultural imports [40]. The effect of nutrients and dietary patterns on human health will take a longer time than that of medications to be observed. In addition, it presents challenges when complying with a certain diet for a long period in huge populations for the study purpose. Therefore, the benefits of an ancient diet such as MedDiet, which is common in particular areas from temporal and geographical perspectives, could be referenced as a compelling healthy eating instance, presenting a similar validity and reliability to the evidence from epidemiologic or interventional studies. Since the ingredients of MedDiet are common and readily available worldwide, governments can advise their population on healthy eating that is suitable to their local context based on the MedDiet pattern.

Cluster 3 (blue) mainly focuses on the importance of overall lifestyle optimization and means of realization. Research shows that diets typically need the assistance of other lifestyles to accomplish and maximize the health benefits. A randomized controlled trial revealed that the time window for eating restrictedly during the day had superior metabolic effects to that at the night [53,54]. Conversely, feeding during the night may shorten lifespans, independent of diet composition and calories [55]. Nowadays, one-third of the world population is haunted by insomnia [56] and the prevalence of late-night eating is highly associated with sleep impairment [57]. Fortunately, exercise proved to be not only a non-pharmacological approach that could improve sleep quality [58] but had synergistic effects with healthy diets [58,59]. e-Health, a set of technologies applied via the internet, provides a viable solution for self-management of daily life in general populations [60]. Based on the big data created via e-Health, a personalized therapeutic algorithm could be used to provide individualized advice on diet, exercise, and sleep in effectively monitoring and adjusting daily lifestyles timely [61,62]. As aforementioned, violating the circadian rhythm undermines the efficacy of healthy diets and thus an overall healthy lifestyle should be promoted. The overall optimization of individual lifestyles might be accomplished or enhanced by eHealth in the future.

Cluster 4 (yellow) mainly focuses on the challenge during the course of healthy eating against obesity. Dietary strategies are critical to fighting against the high prevalence of overweight/obesity accounting for 39% in adults and over 18% among youths [63]. Although the recommended approaches such as low-carbohydrate high-protein diet, ketogenic diet, and intermittent fasting were effective for reducing weight to get started [64,65,66], for the long term, the most effective method is still not identified because different dieting models have promoted similar weight loss [67]. Additionally, all extreme diets against obesity are hard to sustain and caution is required due to safety issues [68,69]. Meanwhile, overweight/obesity among college students [70] is usually overestimated as a result of following the fashion of thinness, slim food recipes, or regimens that are frequently promoted on social media [71,72]. Such prevailing and overwhelming information among the young adults might have resulted in body dissatisfaction and orthorexia nervosa [71]. Orthorexia nervosa, an obsession with “healthy eating” and pure food, was prevalent across countries or populations, ranging from 6.9% to 88.7% [73]. Orthorexia nervosa, an inflexible and extreme diet, has raised growing concerns about physical and mental health, such as nutritional deficits and social exclusion [74]. Along with the global epidemic of obesity, losing weight will become a prevalent topic and vital strategy for public health intervention. To avoid any mistaken consciousness and concept of obesity and preventable side effects related to losing weight, the interventions for weight management should be personalized and administered by multidisciplinary professionals combining nutrition, health, and cognitive-behavioral counseling.

To the best of our knowledge, this is the first bibliometric analysis of healthy eating. Some limitations of the study are worth mentioning. First, we selected the papers published only in English, thus there may be a linguistic bias. The lack of standardization of expression of authors and institutions across publications can also result in an analytic bias. From the perspective of geography, the limited number of African and South American countries that have studied healthy eating may lead to ethnic bias. Second, some of the recently published literature might be discounted because of low citation frequency during the short period of public access. Third, this bibliometric analysis focused on quantitative analysis but not on evaluation and analysis of the quality of the papers. Finally, although the WoS is a large and optimal database for bibliometrics, inevitably, data might not be exhaustive in the WoS. 

## 5. Conclusions

Over the last two decades, there has been a progressive increase in the number of publications and citations on research related to healthy eating across many countries, institutions, and authors. This study sheds light on current trends, global collaboration patterns, basic knowledge, research hotspots, and emerging frontiers of healthy eating. Food insecurity, the Mediterranean diet, lifestyle, and losing weight were the major themes on the healthy eating topic over the last 20 years. Healthy dietary patterns and clinical applications of healthy eating should be the next hotspots in this research field. The findings of the present study will provide valuable guidance for future research and may offer an opportunity to confront three major global challenges.

## Data Availability

All data generated or analyzed during this study are included in this published article.

## Supplementary Materials

The following supporting information can be downloaded at: https://www.mdpi.com/article/10.3390/nu15061461/s1, Figure S1 Distribution and contribution of 32 productive countries/regions globally; Figure S2 the co-citation network map of 69 highly cited journals; Figure S3 The cooperative network map of 153 most productive institutions; Figure S4 The co-authorship network map of 324 most productive authors; Table S1. Top10 most productive countries/regions in healthy eating research; Table S2. Top 10 most productive journals in healthy eating research; Table S3. Top 10 most productive institutions in healthy eating research; Table S4. Top 10 most productive authors in healthy eating research.

## Abbreviations

JCR	Journal Citation Reports;
MedDiet	Mediterranean diet;
NCDs	non-communicable diseases;
TS	topic search;
UPFs	ultra-processed foods;
WoS	the Web of Science
