# Supplementary material for: Global Scientific Trends on Healthy Eating from 2002 to 2021: A Bibliometric and Visualized Analysis
Source: Nutrients. 2023 Mar 17;15(6):1461. doi: 10.3390/nu15061461 (PMC10054585; doi:10.3390/nu15061461)
Supplement: Supplementary file 1 [file nutrients-15-01461-s001.zip › nutrients-2226490-supplementary.pdf]

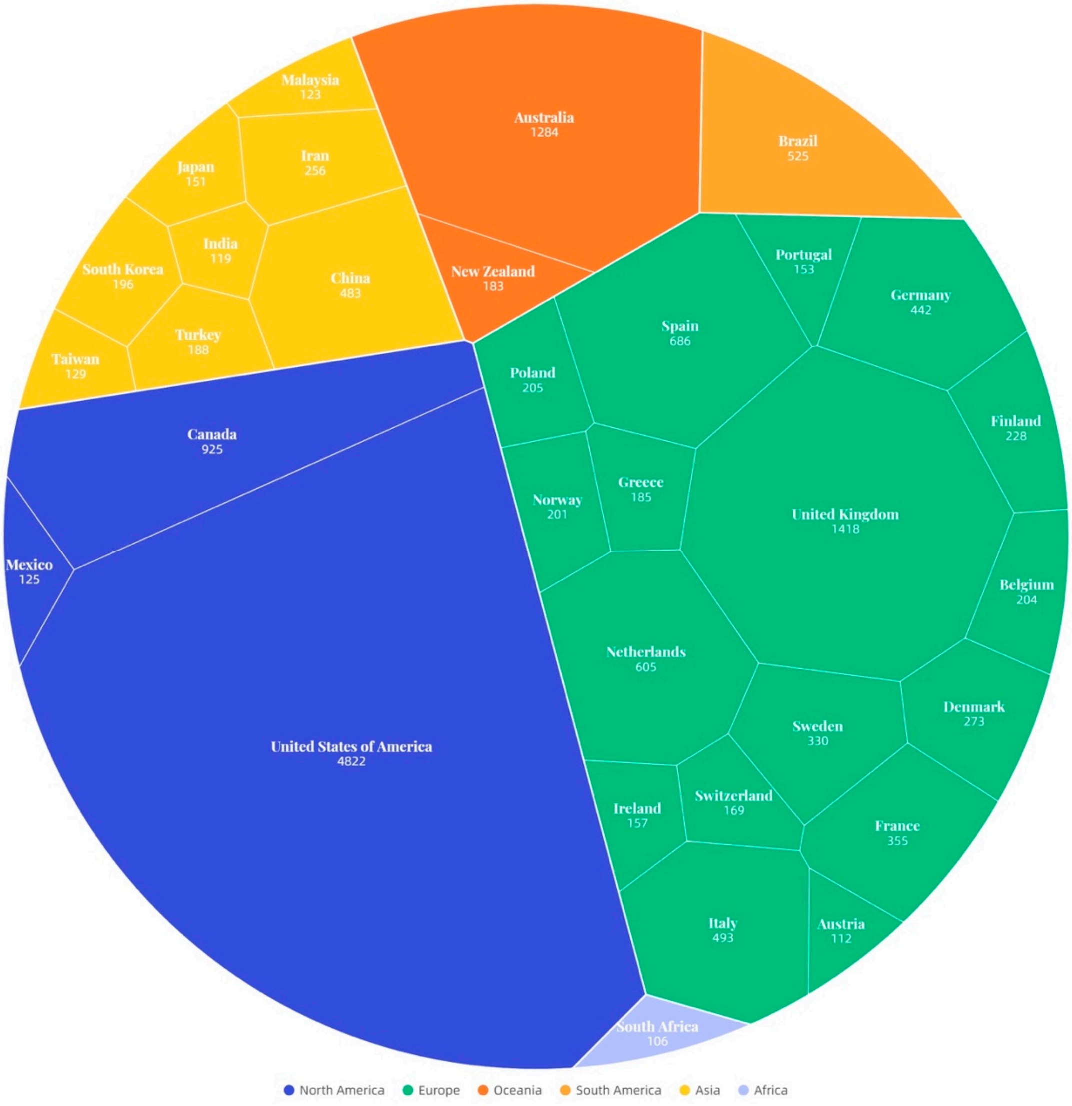

Figure S1 Distribution and contribution of 32 productive countries/regions globally

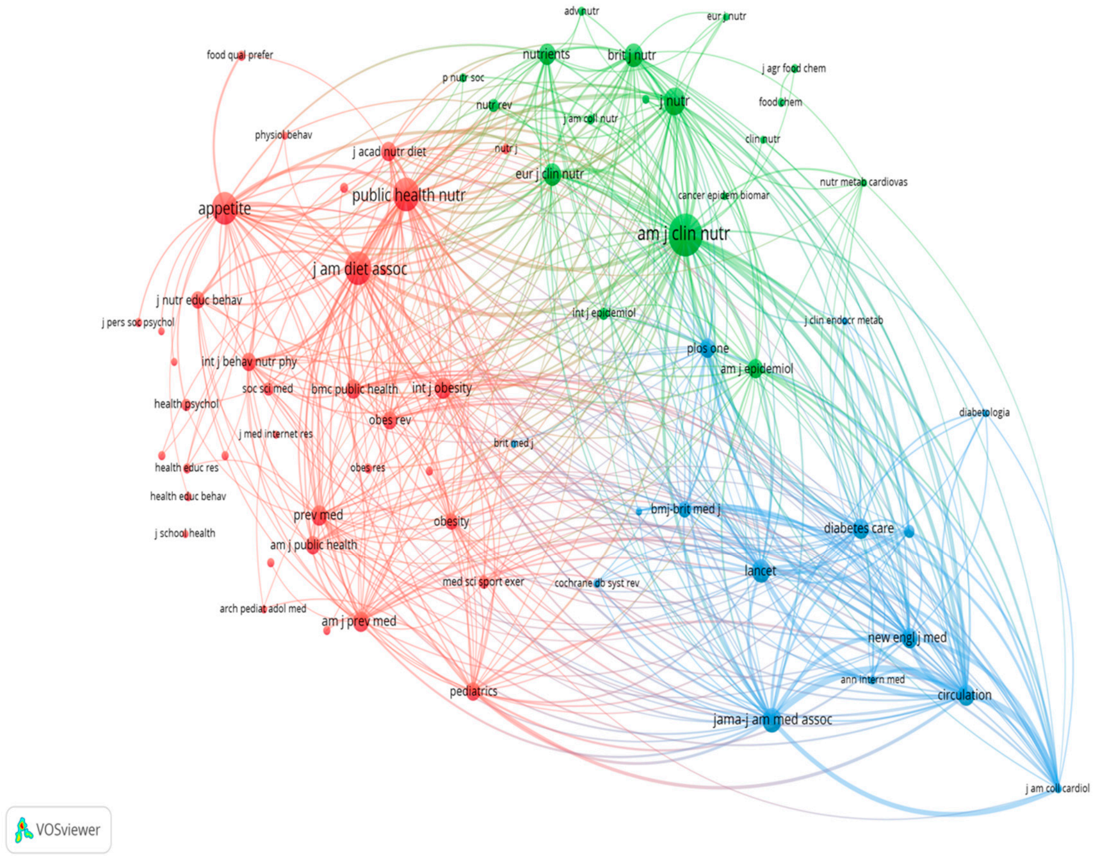

Figure S2 the co-citation network map of 69 highly cited journals

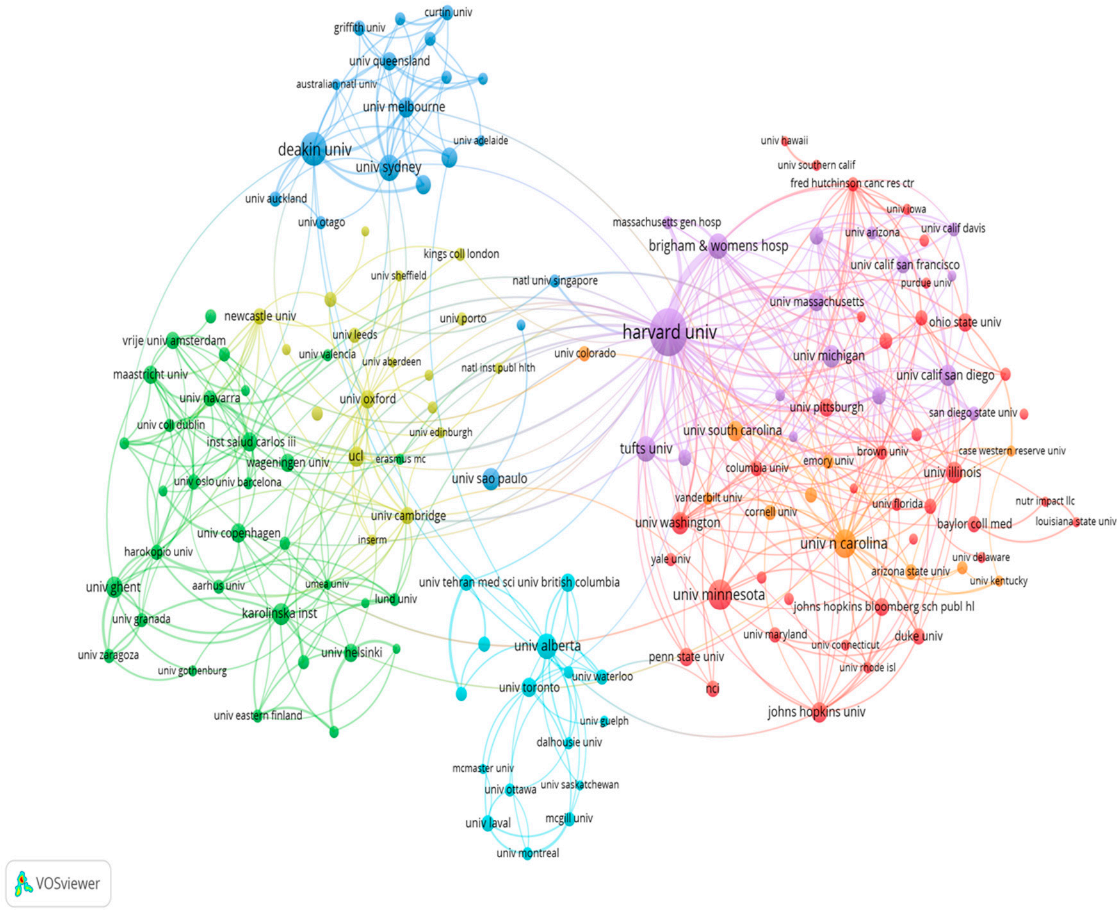

Figure S3 The cooperative network map of 153 most productive institutions

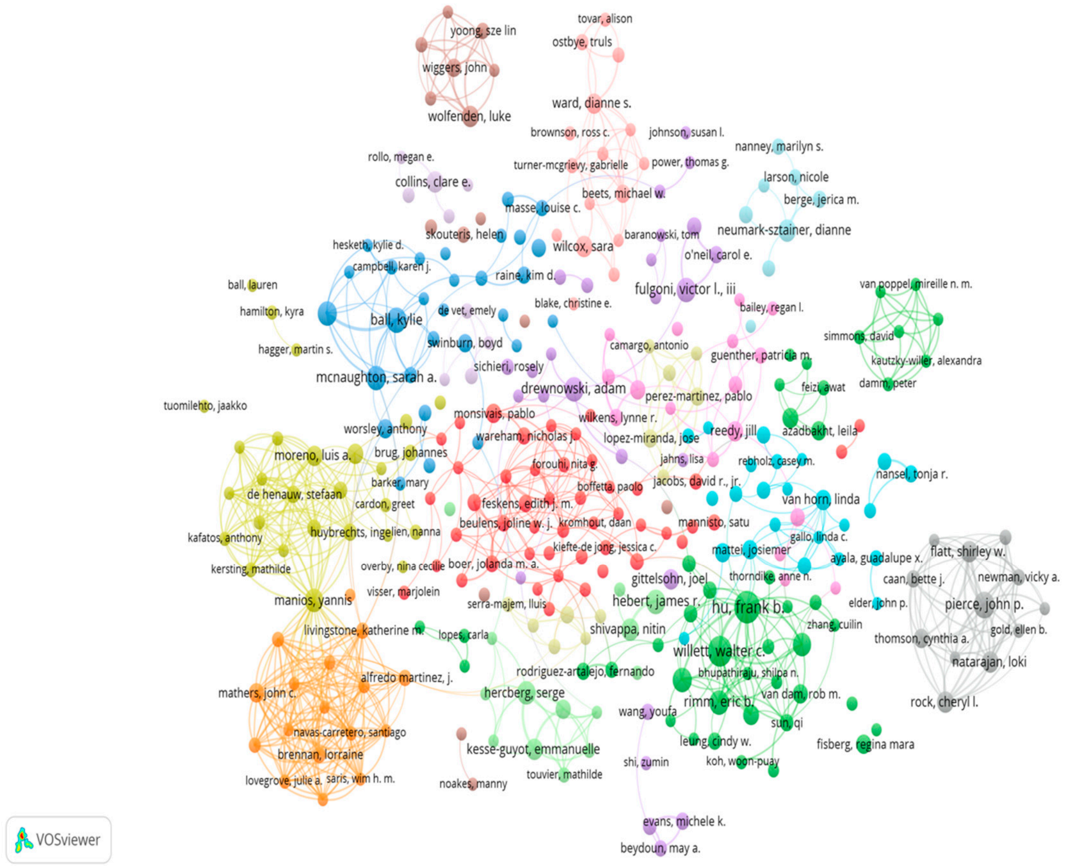

Figure S4 The co-authorship network map of 324 most productive authors

Table S1. Top10 most productive countries/regions in healthy eating research

Rank	Country	Counts	Citations	Avg. Citations	Avg. Pub. Year
1	United States	4,822	129,710	27	2015.44
2	United Kingdom	1,418	48,000	34	2015.12
3	Australia	1,284	33,901	26	2015.85
4	Canada	925	24,743	27	2015.12
5	Spain	686	18,782	27	2016.64
6	Netherlands	605	14,900	25	2016.44
7	Brazil	525	8,827	17	2017.25
8	Italy	493	15,733	32	2016.52
9	China	483	11,270	23	2017.82
10	Germany	442	17,593	40	2016.26

Avg. Citations, average citations; Ave. Pub. Year, average publication year

Table S2. Top 10 most productive journals in healthy eating research

Rank	Journal	IF2020	JCR	Counts	Citations	Avg. Citations	Avg. Pub. Year
1	Nutrients	5.719	Q1	678	5,397	8	2019.36
2	Public Health Nutrition	4.022	Q2	475	11,148	23	2015.39
3	Appetite	3.868	Q1	399	9,304	23	2016.19
4	BMC Public Health	3.295	Q2	362	6,804	19	2015.80
5	Int J Environ Res Public Health	3.390	Q1	240	1,205	5	2019.50
6	American Journal of Clinical Nutrition	7.047	Q1	203	12,121	60	2014.65
7	J Acad Nutr Diet	4.910	Q2	194	5,881	30	2016.75
8	PLOS ONE	3.240	Q2	186	3,701	20	2017.01
9	British Journal of Nutrition	3.718	Q3	182	5,006	28	2014.83
10	Journal of Nutrition	4.798	Q2	177	8,761	49	2014.88

Avg. Citations, average citations; Ave. Pub. Year, average publication year; Int J Environ Res Public Health, International Journal of Environmental Research and Public Health; J Acad Nutr Diet, Journal of The Academy of Nutrition and Dietetics; JCR, Journal Citation Reports; Q, quartile

Table S3. Top 10 most productive institutions in healthy eating research

Rank	Institution	Counts	Citations	Avg. Citations	Avg. Pub. Year
1	Harvard University	477	23,020	48	2016.16
2	Deakin University	271	8,217	30	2014.99
3	University of Minnesota	228	10,183	45	2014.60
4	University of North Carolina	200	5,656	28	2014.84
5	University of Sydney	179	5,848	33	2015.74
6	Brigham & Women's Hospital	168	11,220	67	2015.35
7	Tufts University	167	6,747	40	2016.20
8	University of Alberta	164	4,347	27	2014.65
9	University of Sao Paulo	138	2,122	15	2017.16
10	Karolinska Institute	136	5,481	40	2016.44

Avg. Citations, average citations; Ave. Pub. Year, average publication year

Table S4. Top 10 most productive authors in healthy eating research

Rank	Author	Gender	Counts	Citations	Avg. Citations	Avg. Pub. Year
1	Hu, Frank B.	Male	75	7,526	100	2015.05
2	Willett, Walter C.	Male	65	4,795	74	2015.70
3	Pierce, John P.	Male	48	2,813	59	2010.94
4	Ball, Kylie	Female	44	1,667	38	2013.48
5	Drewnowski, Adam	Male	40	1,184	30	2017.23
6	Mcnaughton, Sarah A.	Female	40	1,288	32	2016.10
7	Crawford, David	Male	39	1,742	45	2012.55
8	Rimm, Eric B.	Male	39	3,159	81	2017.03
9	Fulgoni, Victor L.	Male	38	1,360	36	2014.87
10	Fung, Teresa T.	Female	38	2,851	75	2015.79

Avg. Citations, average citations; Avg. Pub. Year, average publication year
